# Point Mutation of a Non-Elastase-Binding Site in Human α1-Antitrypsin Alters Its Anti-Inflammatory Properties

**DOI:** 10.3389/fimmu.2018.00759

**Published:** 2018-05-01

**Authors:** Yotam Lior, Mariana Zaretsky, David E. Ochayon, Diana Lotysh, Boris M. Baranovski, Ronen Schuster, Ofer Guttman, Amir Aharoni, Eli C. Lewis

**Affiliations:** ^1^Department of Clinical Biochemistry and Pharmacology, Faculty of Health Sciences, Ben-Gurion University of the Negev, Be’er Sheva, Israel; ^2^Department of Life Sciences, Ben-Gurion University of the Negev and National Institute for Biotechnology, Be’er Sheva, Israel

**Keywords:** α1-antitryspin, protein structure, reactive center loop, recombinant protein, pharmacokinetics, inflammation, anti-inflammatory

## Abstract

**Introduction:**

Human α1-antitrypsin (hAAT) is a 394-amino acid long anti-inflammatory, neutrophil elastase inhibitor, which binds elastase *via* a sequence-specific molecular protrusion (reactive center loop, RCL; positions 357–366). hAAT formulations that lack protease inhibition were shown to maintain their anti-inflammatory activities, suggesting that some attributes of the molecule may reside in extra-RCL segments. Here, we compare the protease-inhibitory and anti-inflammatory profiles of an extra-RCL mutation (cys232pro) and two intra-RCL mutations (pro357cys, pro357ala), to naïve [wild-type (WT)] recombinant hAAT, *in vitro*, and *in vivo*.

**Methods:**

His-tag recombinant point-mutated hAAT constructs were expressed in HEK-293F cells. Purified proteins were evaluated for elastase inhibition, and their anti-inflammatory activities were assessed using several cell-types: RAW264.7 cells, mouse bone marrow-derived macrophages, and primary peritoneal macrophages. The pharmacokinetics of the recombinant variants and their effect on LPS-induced peritonitis were determined *in vivo*.

**Results:**

Compared to WT and to RCL-mutated hAAT variants, cys232pro exhibited superior anti-inflammatory activities, as well as a longer circulating half-life, despite all three mutated forms of hAAT lacking anti-elastase activity. TNFα expression and its proteolytic membranal shedding were differently affected by the variants; specifically, cys232pro and pro357cys altered supernatant and serum TNFα dynamics without suppressing transcription or shedding.

**Conclusion:**

Our data suggest that the anti-inflammatory profile of hAAT extends beyond direct RCL regions. Such regions might be relevant for the elaboration of hAAT formulations, as well as hAAT-based drugs, with enhanced anti-inflammatory attributes.

## Introduction

Human α1-antitrypsin (hAAT) is a 52-kDa, 394-amino acid long serum glycoprotein, a member of the serine protease inhibitor superfamily. The molecule is secreted primarily by hepatocytes to the circulation, in both steady state and acute-phase responses (1–3). Additionally, hAAT is produced by lung epithelia, intestinal paneth cells, and M2-like macrophages (4). Several mutations in the gene coding for hAAT have been known to result in significantly low circulating levels of hAAT, a rare genetic condition termed α1-antitrypsin deficiency (AATD). The Z variant (Glu342Lys) is the most common variant of AATD, followed by the S variant (Glu264Val) (5). AATD is most commonly associated with early-onset non-smoker lung emphysema as well as liver cirrhosis, vasculitis, and bacterial pneumonia (5–8). Being a one-gene disease, several research teams have been making advances in the field of hAAT gene-therapy, primarily *via* an adenoviral backbone construct (clinical trials NCT01054339, NCT02168686, NCT00377416, and NCT00430768) (9, 10), the only standard of care for AATD at present involves life-long weekly infusions of affinity-purified human plasma-derived AAT, aimed at restoring circulating hAAT levels (7, 11).

While globular in structure, hAAT has a reactive center loop (RCL, positions 357–366) that protrudes from its surface, and that acts as a sequence-specific bait for serine-proteases (2, 12), among which are neutrophil elastase, cathepsin G, and proteinase-3 (13, 14). RCL cleavage leads to the covalent attachment of the targeted protease to hAAT, followed by a conformational change and the removal of the hAAT:protease complex from the circulation (14).

Interestingly, proteases outside the serine-protease family are also inhibited by hAAT, albeit to a lesser extent. These include metalloproteases [e.g., MMP-9 (15–17), ADAMTS-4 (18), and cysteine-proteases (e.g., caspase-3) (19, 20)], suggesting that some functions of the molecule may extend beyond the specificity conferred by the primary sequence of the RCL. As such, it has been proposed that the globular surface of hAAT may contain significant functional attributes. Indeed, it has been established that hAAT directly binds IL-8 (21, 22), as well as to polymeric immunoglobulin receptor (23), gp96 (24), HSP70 (25), oxidized cholesterol within lipid rafts and serum lipids (26–29), HDL particles (30–32), and LRP1 receptor (33). Furthermore, certain activities that are attributed to hAAT appear to be reproducible in formulations that *lack* elastase inhibition as in the case of recombinant Fc-hAAT and truncated hAAT (26, 34–36).

The breadth of anti-inflammatory and immunomodulatory functions of hAAT has gained increased recognition in the past decade. hAAT promotes production of anti-inflammatory cytokines, such as IL-10 (37) and IL-1 receptor antagonist (IL-1Ra) (38), and inhibits the release of pro-inflammatory cytokines and chemokines, such as IL-6 and TNFα (3, 39–43). In the context of allograft transplantation, hAAT modifies dendritic cell responses (37, 44) and B lymphocyte activities (45), reduces the levels of inducible co-stimulatory molecules, e.g., CD40 and CD86, and promotes regulatory T cell expansion (4, 41, 46). Of particular interest, hAAT reduces soluble TNFα levels (42, 43) and interferes with TNFα-dependent responses. Inducible membrane-associated TNFα appears to accumulate on the surface of hAAT-treated leukocytes (47), even though TNFα cleavage requires ADAM metallopeptidase domain 17 (ADAM17/TACE) (43), which is outside the repertoire of hAAT protease inhibition.

Mutations within the RCL usually alter hAAT protease-inhibiting specificity or total protease-inhibiting capacity, as reported with regards to a mutation in which proline is substituted with cysteine within the RCL region (pro357cys) (48). However, little is known regarding the effect of such mutations in as far as the anti-inflammatory properties of hAAT are concerned. Furthermore, only a few studies explored non-AATD-causing mutations *outside* the RCL in terms of anti-proteases and anti-inflammatory effects.

In the present study, we revisited a previously described intra-RCL mutation (pro357cys) known to lack anti-protease activities (48). To better understand the effects of this intra-RCL mutation, we compared its anti-inflammatory attributes to those of wild-type (WT) hAAT as well as to those of novel intra-RCL (pro357ala) and extra-RCL (cys232pro) hAAT variants. Indeed, the functions of hAAT evaluated hereby, appear to extend beyond protease-inhibition and include both *in vitro* and *in vivo* anti-inflammatory activities.

## Materials and Methods

### Plasmid Constructs

Human AAT EST clone was purchased from Open Biosystems (GE Healthcare, Chicago, IL, USA) and amplified by PCR using FW 5′-GATCACCG-GTGAATTCGATATCTCGAGCACCATGGTTATGCCGTCTTCTGTCTCGTGGGGCATCC-3′ and RE 5′-GCTGGGCAAGGTGGGCACTCCACAGATCTCTACTA-GTGATGGTGATGATGATGATGATGTTTTTGGGTGGGATTCACCAC-3′ primers. A His-tag sequence was added to the C terminal. Specific mutations for the replacement of C232 and P357 were inserted by assembly PCR using the primers: FW-AAT-C232P TTTAGGCATGTTTAACATC-CAGCACCCCAAGAAGCTGTCCAGCTGGGTGCTGCTG and RE-asm-AAT GTGCTGGATGTTAAACATGCCTAAACG for C232P, FW-asm-PtoC-AAT TTAGAGGCCATATGCATGTCTATCCCCCCCGAGG and RE-asm-PtoC-AAT CCTCGGGGGGGATAGACATGCATATGGCCTCTAA for P357C, and FW-asm-PtoA-AAT GTTTTTAGAGGCCATAGCCATGTCTATCCCCCCCGAG and RE-asm-PtoA-AAT CTCGGGGGGGATAGACATGGCTATGGCCTCTAAAAAC for P357A. Sequences were cloned into pFUSE plasmid (Invivogen, San Diego, CA, USA) using NEBuilder HiFi DNA Assembly Master Mix (New England Biolabs, Ipswich, MA, USA), according to manufacturer’s instructions. Naïve human AAT signal peptide was used in protein expression. Plasmids were replicated in *E. coli* (HIT Competent Cells-DH5α, Real Biotech Corporation, Banqiao city, Taiwan) and purified using Wizard^®^ Plus SV Minipreps DNA Purification Systems (Promega, Fitchburg, WI, USA), according to manufacturer’s instructions.

### Recombinant Protein Production and Purification

HEK-293F cells (CRL-1573, ATCC, Manassas, WV, USA) were cultured in FreeStyle 293 expression medium (Invitrogen, Carlsbad, CA, USA) in 8% CO_2_ shaking incubator. Cells were transfected using GeneTran™ transfection reagent (Biomega, San Diego, CA, USA) according to manufacturer’s instructions. Six days post-transfection, supernatants were collected and secreted hAAT was purified using Ni beads (Calbiochem, Merck Millipore, Darmstadt, Germany) by standard protocol. After protein purification, samples were assessed for purity and molecular weight on a 10% polyacrylamide gel stained with coomassie brilliant blue; commercial clinical-grade serum-purified hAAT (Glassia, Kamada, Ness-Ziona, Israel) was used as reference. Protein concentrations were determined using micro-volume spectrophotometer (Nanodrop, ThemoFisher Scientific, Waltham, MA, USA) and Bradford Protein Assay (Bio-Rad Laboratories, Rishon-LeZion, Israel).

### Neutrophil Elastase Activity Assay

Neutrophil elastase activity was determined in acellular conditions using a designated kit (Sigma-Aldrich, Lois, MO, USA), according to manufacturer’s instruction (final elastase concentration per well: 0.39 μM). rhAAT variants were pre-incubated with the commercial enzyme prior to kinetic evaluation of color-producing substrate processing.

### Mice

C57BL/6 mice (6–8 weeks old males and females from Harlan Laboratories Ltd., Jerusalem, Israel) were used for all experiments. The study was approved by the Ben-Gurion University of the Negev Animal Care and Use Committee.

### Production of Bone Marrow-Derived Macrophages (BMDM)

The tibia and femur of C57BL/6 mice were surgically removed and thoroughly flushed through a 70-μM sterile nylon cell strainer (Falcon; BD Biosciences Discovery Labware, San Jose, CA, USA) with PBS (Biological Industries, Beit Ha’emek, Israel). Cells were resuspended and cultured in 10 ml complete RPMI 1640 (containing 10% fetal bovine serum, 50 U/ml streptomycin/penicillin, 50 μg/ml l-glutamine, all from Biological Industries), 50 μM β2-mercaptoethanol (Sigma-Aldrich, Rehovot, Israel) and 20 ng/ml recombinant Granulocyte Macrophage Colony-Stimulating Factor (rGM-CSF, PeproTech, Rocky Hill, NJ, USA). Medium containing rGM-CSF was added on day 3 and on day 6. Cell populations were confirmed as being >95% CD11b^+^ after 9 days of incubation with rGM-CSF by flow cytometry.

### Thioglycolate-Elicited Primary Peritoneal Cells

C57BL/6 mice were injected with thioglycolate (3% v/v, Sigma-Aldrich; i.p., 1.5 ml per mouse). Five days later, peritoneal lavage was performed with cold PBS. Recovered cell suspensions were filtered through a 70-μM sterile nylon strainer. Cells were then resuspended in complete RPMI 1640. Cell cultures were routinely verified to be >95% CD11b^+^/F4-80^+^ cells by flow cytometry.

### Cell Activation Assays and Flow Cytometry

Peritoneal macrophages and BMDMs, as indicated, were seeded at 2–3 × 10^5^ cells per well in 300 μl complete RPMI 1640. Recombinant hAAT variants were added at indicated concentrations for overnight incubation. Cells were then carefully washed with PBS and medium was replaced with the same concentrations of rhAAT variants, as well as LPS (Sigma-Aldrich) at indicated concentrations. Twenty-four hours later, supernatants were collected and analyzed for IL-6 and TNFα concentrations using specific ELISA (Biolegend, San Diego, CA, USA).

Cells were gently removed with a rubber policeman and suspended in FACS buffer (PBS containing 1% BSA from Biological Industries, 0.1% sodium azide and 2 mM EDTA, both from Sigma-Aldrich). Blocking was performed at room temperature for 20 min using anti-CD16/32 antibody (Biolegend). Staining was performed at 4°C for an additional 20 min using the following anti-mouse antibodies: anti-CD40-FITC (3/2.3), anti-CD86-PE (GL-1), anti-TNFα-APC (MP6-XT22), anti-CD11b-Pacific blue (M1/70), all from Biolegend, and anti-F4/80-PerCP-Cy5.5 (BM8.1) (Merc, Temecula, CA, USA). Fluorescent readout was determined using BD Canto II and data were analyzed by FLOWJO 10.0.8r1 software (Flowjo, LLC Data Analysis Software, Ashland, OR, USA). After exclusion of cellular debris and duplicated cells, F4-80^+^/CD11b^+^ population was selected and surface expression levels of CD40 and CD86 were assessed and compared between samples.

### *In Vivo* LPS-Induced Peritonitis

Mice were pretreated with 100 μl of PBS or rhAAT variants (50 μg per mouse i.p., *n* = 20 per experiment) for 3 h, then treated with 1 mg/kg LPS (i.p.). Blood samples (20 μl) were collected from the tail vein at 1.5, 3, and 24 h later, and serum was separated by centrifuge; sera were analyzed for TNFα concentrations using specific ELISA (R&D Systems).

### Real-Time Quantitative PCR

RAW264.7 cells (TIB-71, ATCC) were seeded at 5 × 10^5^ cells per well in 500 μl complete RPMI 1640. Cells were carefully washed and medium replaced with identical concentrations of rhAAT variants and LPS at indicated concentrations. Total RNA was purified at 1, 3, and 6 h poststimulation using total RNA purification kit (Norgen, Thorold, ON, Canada), according to manufacturer’s instructions. Sample concentrations were normalized to RNA content using micro-volume spectrophotometer (Nanodrop) and then reverse-transcribed with qScript cDNA synthesis kit (Quanta Biosciences, Gaithersburg, MD, USA), according to manufacturer’s instructions. cDNA amplification was performed and gene transcription was analyzed by qPCR (StepOnePlus real-time PCR system, ThemoFisher Scientific) using the following primers: 18S FW 5′-TCAACACAGGGATCGGACAACACA-3′ RE 5′-GCCTTGGATCAAGTTCACAGGCAA-3′; TNFα FW 5′-CCCACGTCGTAGCAAACCAC-3′ RE 5′-CCCTTGAAGAGAACCTGGGAG-3′.

### Pharmacokinetics Study

rhAAT variants were introduced into mice (50 μg/mouse, i.v.). Blood samples (40 μl) were collected from the tail vein and circulating serum hAAT levels were determined at 1, 12, and 24 h from injections using species-specific hAAT ELISA (ICL Lab, Portland, OR, USA). T_0.5_ and distribution volume were calculated using PKsolver add-in for Microsoft Excel (49).

### Statistical Analysis

Two-tailed Mann–Whitney test was used to assess differences between selected experimental conditions. Results are expressed as mean ± SEM, *p* ≤ 0.05 was considered significant. All statistical analyses were performed using GraphPad Prism version 6.01.

## Results

### Recombinant hAAT Variants

**Figure 1:**
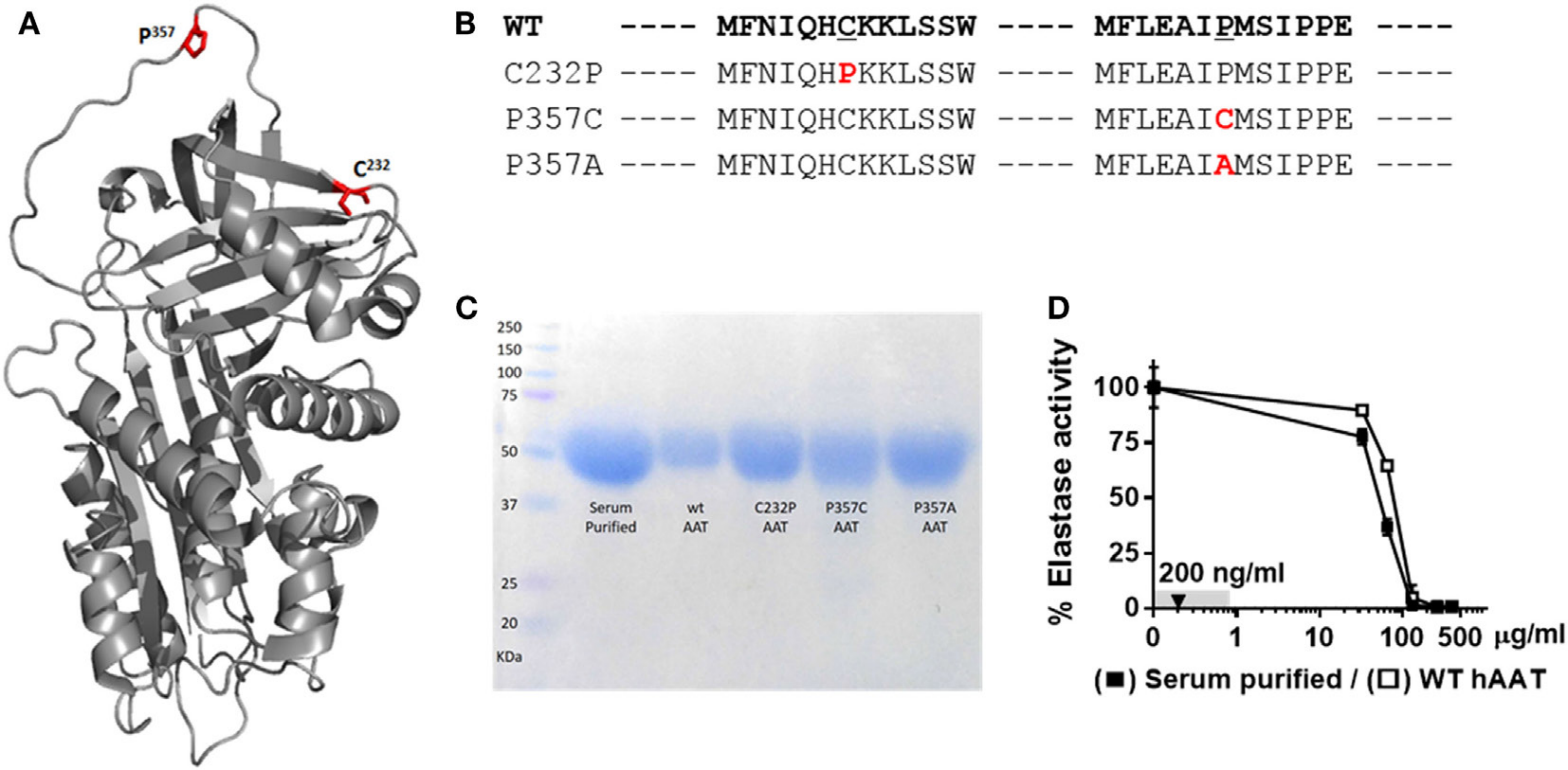
α1-Antitrypsin. **(A)** Computerized 3D model of AAT. *Red*, mutagenesis specific loci, C^232^ and P^357^. **(B)** FASTA sequence of WT-hAAT and three generated mutations. *Red*, mutation sites. **(C)** Representative Commassie brilliant blue blot of purified rhAAT variants and serum-purified human α1-antitrypsin (hAAT). **(D)** Inhibitory potency of WT-hAAT and serum-purified hAAT over neutrophil elastase (0.39 μM). *Gray*, concentrations range used in this study. *Triangle*, concentration used in anti-inflammatory studies. Data representative of three independent experimental repeats.

Mutations at amino acid positions 357 (*inside* the RCL) and 232 (*outside* the RCL) were generated, as illustrated in Figures 1A,B; for this, HEK-T293F cells were transfected with respective plasmid constructs and allowed to release His-tag WT recombinant hAAT (WT-rhAAT) and its mutated variants (C232P, P357C and P357A). hAAT variants were then affinity-purified, and their size confirmed to be consistent with serum-purified commercially available clinical-grade human AAT (Figure 1C). According to neutrophil elastase inhibition assays (Figure 1D), WT-rhAAT inhibition profile appears consistent with that of serum-purified hAAT, requiring concentrations in the range of micrograms (in contrast to the later experimental 200 ng/ml concentration range, *arrow*). The variants C232P (CP), P357C (PC), and P357A (PA) failed to inhibit neutrophil elastase at all tested concentrations (not shown); expectedly, inhibition of ADAM17 in an acellular inhibition assay was negative for all formulations of rhAAT, including WT-rhAAT (not shown).

### Anti-Inflammatory Attributes of rhAAT Variants at Below Protease-Inhibitory Concentrations

**Figure 2:**
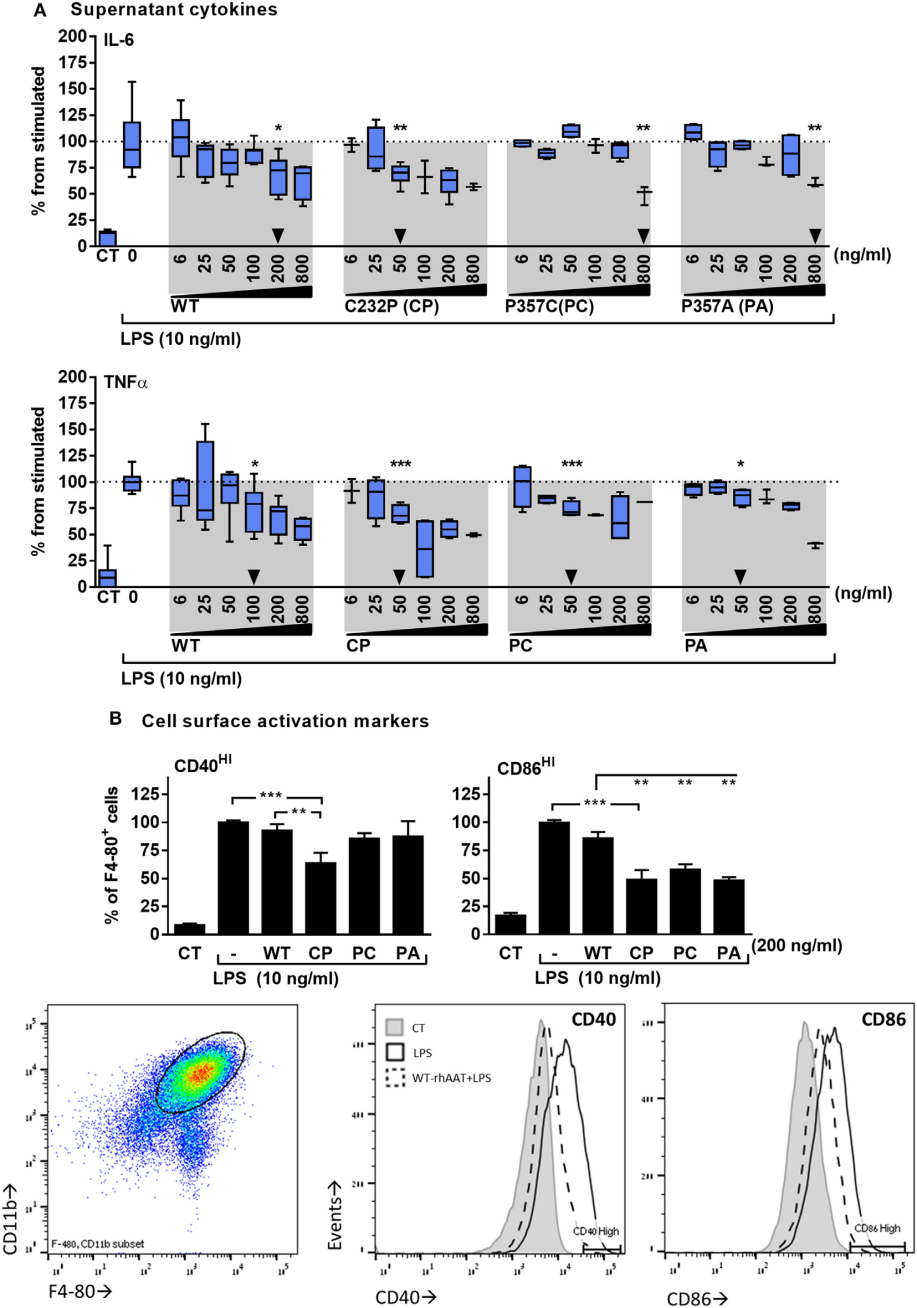
rhAAT anti-inflammatory potency variations on leukocyte LPS-responses. **(A)** Cytokine release, bone marrow-derived macrophages (BMDM) (2 × 10^5^ per well) overnight incubation with complete medium containing indicated doses of rhAAT followed by PBS wash and re-incubation with complete medium containing LPS (10 ng/ml, 24 h). Supernatants analysis for IL-6 and TNFα concentrations by specific ELISA. *Triangle*, first statistically significant difference from stimulated sample. **(B)** Membranal activation markers, BMDM (5 × 10^5^ per well) overnight incubation with complete medium containing 200 ng/ml rhAAT, followed by LPS addition (10 ng/ml, 24 h). Flow cytometric analysis for CD40^HI^ and CD86^HI^. Gate, CD11b^+^/F4-80^+^ cells. Data representative of four to five independent experimental repeats. Mean ± SEM, *p < 0.05, **p < 0.01, ***p < 0.001.

The response of primary murine BMDMs to LPS was tested in the presence of hAAT variants. As shown in Figure 2, the cellular response to LPS included inducible IL-6 and TNFα release (Figure 2A), and increased expression levels of surface CD40 and CD86 (Figure 2B). WT-rhAAT pretreatment at 200 ng/ml resulted in a significant reduction of inducible IL-6 level (31.5% from LPS alone). While PC and PA pretreatment at the same concentration failed to achieve a statistically significant reduction in IL-6 (7 and 12.5%, respectively), CP achieved a significant inhibition of inducible IL-6 levels at concentrations as low as 50 ng/ml (Figure 2A, *arrowhead*). Inducible TNFα supernatant levels displayed a different pattern to that of inducible IL-6. WT-rhAAT pretreatment reached a significant decrease at 100 ng/ml, while CP, PC and PA caused a comparable decline at 50 ng/ml (24.9, 30.4, 25.9, and 14.1%, respectively).

Based on these observations, the concentration of 200 ng/ml was used for evaluating the effect of rhAAT variants on CD40 and CD86 surface expression (Figure 2B). As shown, changes in CD40 and CD86 displayed a pattern similar to that of released inflammatory cytokines: at 200 ng/ml, WT-rhAAT was ineffective in reducing CD40^HI^ or CD86^HI^ cell population proportions, while CP pretreatment resulted in significant reduction in CD40^HI^ and CD86^HI^ cell populations (36 and 51%, respectively). CD86 was responsive to PC and PA, exhibiting a reduction of 42 and 51%, respectively, as opposed to CD40 (14 and 13%, respectively).

In the absence of LPS, WT-rhAAT did not elevate IL-6 and TNFα supernatant levels nor the expression of CD40 and CD86 compared to non-exposed cells (data not shown).

**Figure 3:**
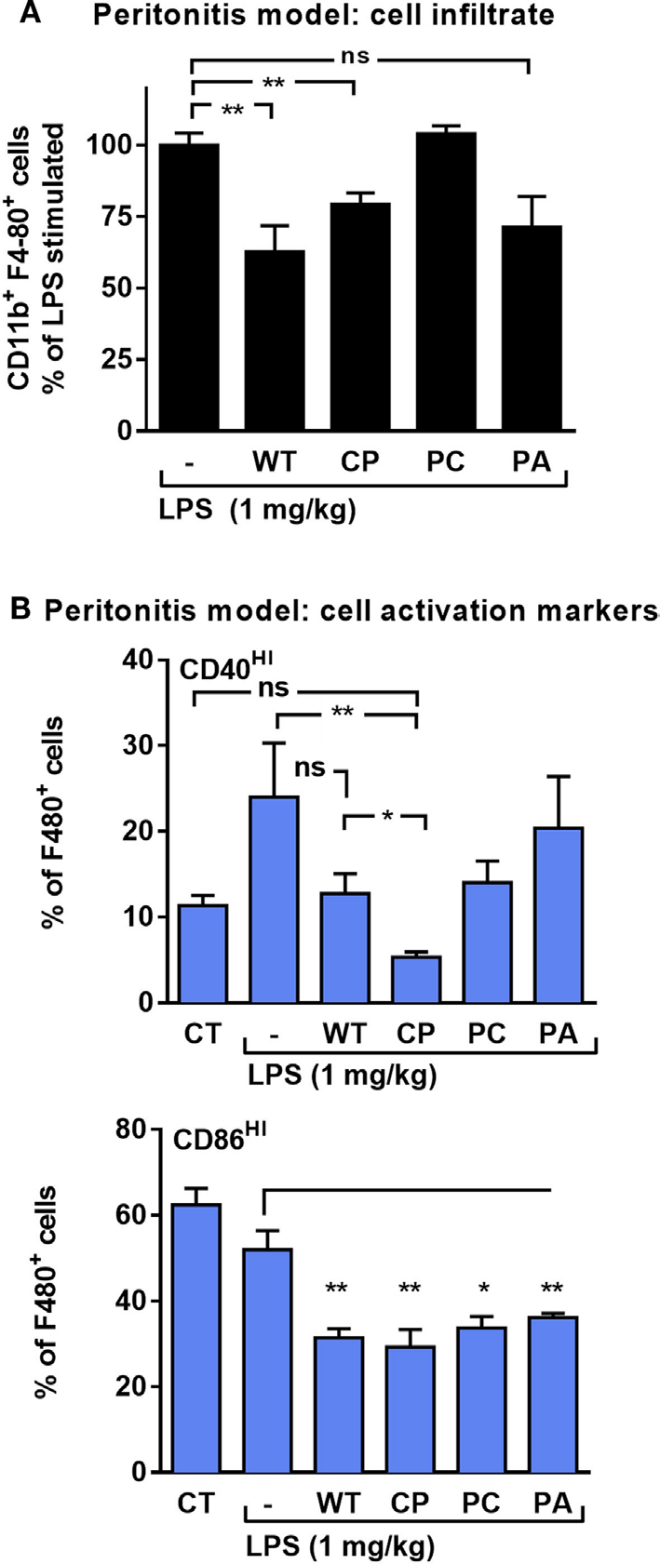
rhAAT anti-inflammatory potency variations in sterile peritonitis *in vivo* models. C57BL/6 mice (*n* = 5 per group) injected with rhAAT (50 μg per mouse) i.p. and 3 h afterward, LPS (1 mg/kg). Peritoneal lavage performed 24 h post LPS injection. Flow cytometric analysis for **(A)** CD11b^+^ F4-80^+^, % of LPS-stimulated **(B)** CD40^HI^ and CD86^HI^, Gate, CD11b^+^ F4-80^+^. CT, PBS injection. Mean ± SEM, *p < 0.05, **p < 0.01 compared to LPS-stimulated group.

*In vivo*, the effect of rhAAT on leukocyte responses to LPS was evaluated in a peritoneal LPS-induced sterile inflammatory model. In this model, activated infiltrating monocytes are readily depicted upon peritoneal lavage. Here, monocytes were characterized by staining for F4-80 and CD11b and then further tested for the proportion of co-stimulatory activation. As shown in Figure 3A, animals pretreated with WT-rhAAT exhibited a 36% reduction in CD11b^+^ F4-80^+^ cell population size compared to the LPS group (set at 100%). While pretreatment with CP or PA led to a 21 and 29% reduction in elicited CD11b^+^ F4-80^+^ cell population, respectively, pretreatment with PC was ineffective in altering cell subtype ratio. The degree of activation of CD11b^+^ F4-80^+^ cells (Figure 3B) depicted a rise in LPS-induced CD40^HI^ cells to 24% of CD11b^+^ F4-80^+^ cells, while pretreatment with WT-rhAAT resulted in a reduction of the CD40^HI^ population to 13% of CD11b^+^ F4-80^+^ cells, similar to the 12% observed by PC pretreatment. In contrast, pretreatment with CP resulted in a greater decline in the proportion of CD40^HI^ cells to 5% of the LPS group, while PA was ineffective in altering the inducible profile of CD40^HI^ on cells. Compared to control untreated animals, CD86^HI^ cell population size was unaffected by *in vivo* LPS stimulation. Nonetheless, significant reductions in CD86^HI^ cell population size were observed under pretreatment with WT, CP, PC and PA rhAAT (31, 29, 34, and 34%, respectively).

### Unique Pharmacokinetics of the CP Variant

**Figure 4:**
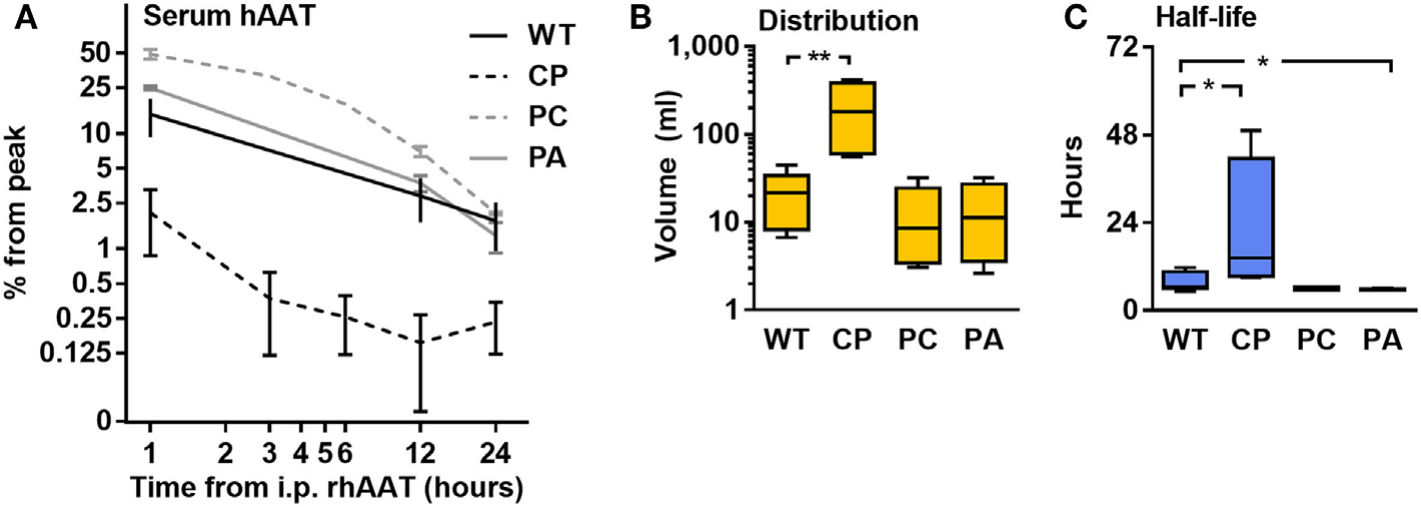
Pharmacokinetics. C57BL/6 mice (*n* = 5 per group) were injected with rhAAT i.v. human α1-antitrypsin (hAAT) concentration analysis by species-specific ELISA from serum samples (1, 12, 24 h). **(A)** hAAT serum concentrations, Mean ± SEM. **(B)** Calculated distribution volume. **(C)** Calculated half-life time. Data representative of three independent experimental repeats., *p < 0.05, **p < 0.01 compared to wild-type (WT).

Half-life and distribution volume for each rhAAT variant were calculated based on time-dependent circulating hAAT concentrations, as determined in mice injected with each rhAAT variant (50 μg, i.v.). As shown in Figure 4, the kinetics of the circulating recombinant forms appears uniform between WT, PC, and PA. However, the levels of circulating CP were 7.13 ± 0.08-fold lower than in WT-rhAAT at as early as 1 h after injection (Figure 4A). Accordingly, its distribution volume was calculated to be 9.5 ± 3.0-fold greater than that of WT-rhAAT (Figure 4B), and its half-life significantly extended (Figure 4C).

### TNFα Expression, Production, and Release

**Figure 5:**
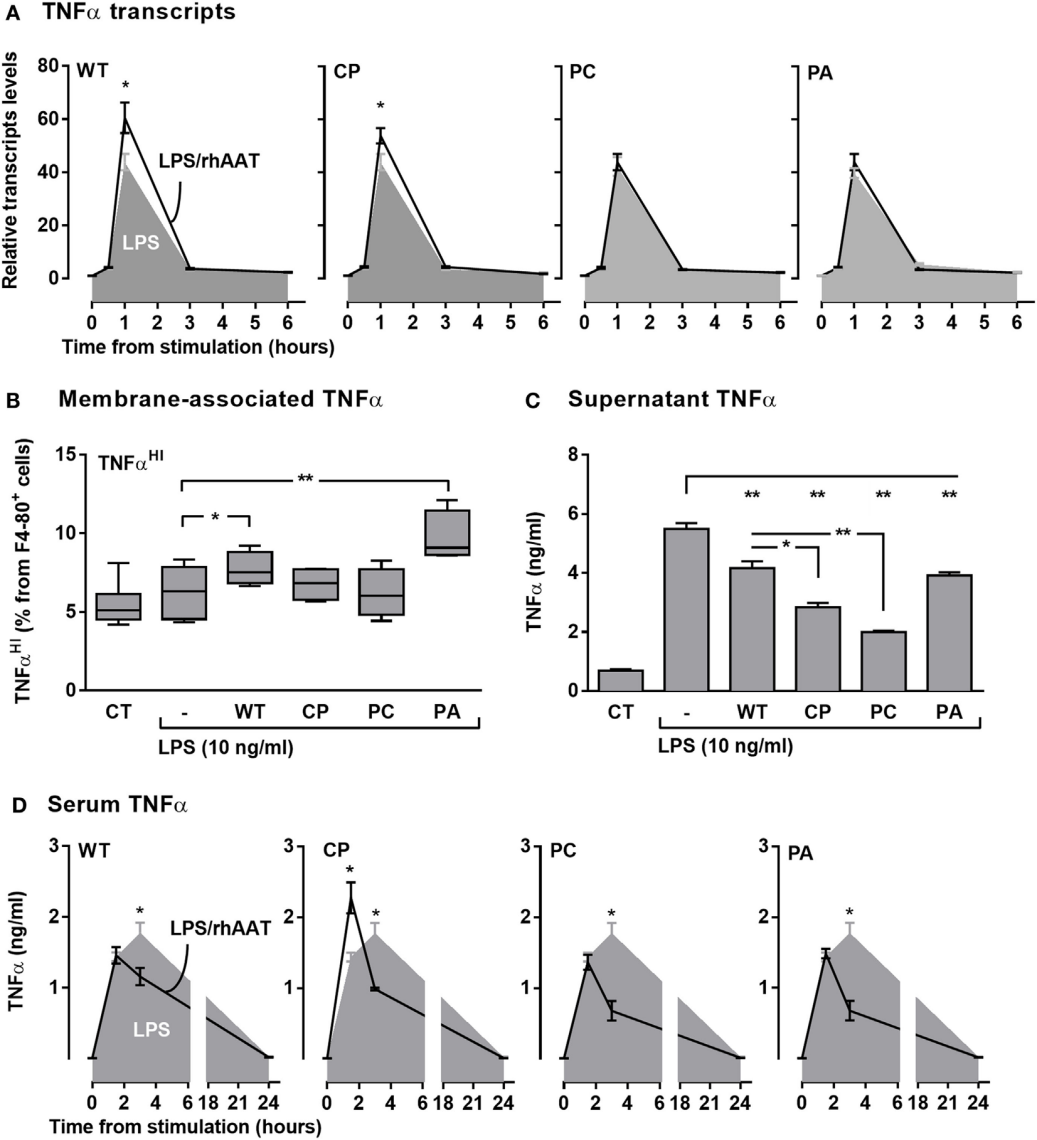
rhAAT effect potency variation on TNFα transcription, expression, and release. **(A)** TNFα transcription, Raw 264.7 cells (0.5 × 10^6^ per well) overnight incubation with complete medium containing 200 ng/ml rhAAT, followed by LPS addition (10 ng/ml, 24 h), nucleic acids extracted (0.5, 1, 3, 6 h) and TNFα transcription assessed by qPCR. Results presented as fold from control. **(B,C)** Peritoneal macrophages (3 × 10^5^ per well) overnight incubation with complete medium containing 200 ng/ml rhAAT, followed by LPS addition (10 ng/ml, 24 h). **(B)** Flow cytometric analysis for membrane-associated TNFα. Gate, CD11b^+^. **(C)** Supernatant TNFα analysis by specific ELISA. **(D)** *In vivo* sterile peritonitis model serum TNFα levels. C57BL/6 mice (*n* = 5 per group) injected with PBS or rhAAT (50 μg/mouse) i.p. and 3 h afterward, LPS (1 mg/kg). TNFα serum analysis by ELISA (1.5, 3, and 24 h). CT, non-stimulated cells. Mean ± SEM. Data representative of two independent experimental repeats. *p < 0.05, **p < 0.01 Compared to LPS-stimulated group.

LPS-stimulated RAW264.7 cells were pretreated with rhAAT variants and several aspects of TNFα expression were determined (Figure 5A *transcription*, Figure 5B *membrane-associated*, Figures 5C and 5D *soluble form* in supernatant and serum *in vivo*, respectively). As shown, LPS had induced a spike in relative TNFα transcript levels (Figure 5A, *shaded*); accordingly, LPS-treated cultured primary peritoneal cells displayed a rise in TNFα release (Figure 5C). Animals injected with LPS exhibited a time-dependent rise in serum TNFα levels (Figure 5D, *shaded*). Membrane-associated TNFα levels (Figure 5B) displayed no significant change upon LPS stimulation, agreeing with the anticipated dynamic of ADAM17-dependent cleavage of membrane-associated TNFα during inflammatory conditions. Unexpectedly, pretreatment with WT-rhAAT resulted in a significant rise in relative TNFα transcript levels 1-h poststimulation (1.53 ± 0.12-fold from LPS alone, Figure 5A), coupled with a decline in serum TNFα levels 3 h poststimulation (1.5 ± 0.02-fold lower than LPS alone, Figure 5D).

Interestingly, while the three rhAAT variants displayed no significant effect on LPS-induced TNFα transcription levels (Figure 5A), pretreatment with CP resulted in a significant earlier narrow spike in serum TNFα levels (Figure 5D). PC displayed a pattern of inhibition similar to that of WT-rhAAT, and PA did not exert a significant effect on LPS-stimulated TNFα transcript levels nor on serum levels. *In vitro* (Figure 5C), the levels of released TNFα levels were consistent with *in vivo* findings in that treatment with WT, CP, PC, and PA caused a decline in soluble TNFα concentrations, most effectively by CP and PC variants.

Expression of TNFα without the emergence of its soluble form could be caused by inhibition of ADAM17 activity (43); such a process is expected to result in elevated levels of membrane-associated non-cleaved TNFα. As shown in Figure 5B, membrane-associated TNFα levels were evaluated after pretreatment with each of the rhAAT variants. Pretreatment with WT-rhAAT resulted in a significant increase in membrane-associated TNFα, corresponding to 24% lower soluble TNFα levels under the same conditions (Figure 5C). Membrane-associated and soluble TNFα levels responded differentially to the various variants; while the membranous effect of CP and PC seemed to be minimal, soluble TNFα levels were reduced by 48 and 63%, respectively. Interestingly, PA was the sole variant which resulted in a significant rise in membrane-associated TNFα levels. However, this rise was coupled with only a 29% decrease in soluble TNFα level, similar to the change observed under WT-rhAAT pretreatment. The overall effect of CP pretreatment, compared to WT-rhAAT pretreatment, appears to involve a minor shift in transcript levels and rapidly declining soluble TNFα levels with only a minimal change in membrane-associated TNFα levels; at the same time, the first evidence of circulating TNFα *in vivo* is pushed up to the 1-h region, temporarily exhibiting serum TNFα levels *higher* than those encountered in LPS treatment alone.

## Discussion

Recent years have witnessed an expansion of potential clinical applications for hAAT treatment beyond that of straightforward augmentation therapy for genetic hAAT deficiency; these include type 1 diabetes (37, 50–52), allogeneic and xenogeneic transplants (44–46, 53, 54), graft-versus-host disease (53, 55, 56), acute myocardial infarction (57–59), inflammatory bowel disease (52, 60), rheumatoid arthritis (61–63), multiple sclerosis (41), and osteoporosis (64, 65). Collectively, these represent an extension of earlier preclinical studies that portray hAAT as possessing anti-inflammatory and immunoregulatory properties (3, 4, 26, 35).

Unexpectedly, a major part of the anti-inflammatory and immunoregulatory properties of hAAT were shown to be independent of protease inhibition (34–36). Evidence for several molecular binding partners is presently on the rise, supporting the possibility that the globular surface of hAAT is relevant to its anti-inflammatory functions. It is, therefore, timely that the functionality of molecular attributes outside the protease inhibitory region of hAAT be investigated. Such an effort coincides with three decades of attempts to generate straightforward clinical-grade recombinant WT-hAAT for AATD augmentation therapy (36, 66–76). The relevance of non-RCL hAAT segments has been addressed in the past primarily in the context of aberrant hAAT aggregation in AATD (14); little attention is drawn to any specific functional significance to non-RCL segments in the molecule. Thus, we suggest that the development of recombinant hAAT may benefit from a better understanding of its non-RCL-related biology, as it may host biologically important anti-inflammatory and immunomodulatory activities.

This study examines the structure–function relationships of hAAT using recombinant proteins. While recombinant proteins are known to vary from their biological counterparts in multiple aspects, e.g., posttranslational modifications, it is important to note that *native* hAAT varies between individuals, particularly with regards to their glycosylation patterns (1, 66, 67, 77). Moreover, factory release criteria for clinical-grade hAAT do not require structural homogenicity, introducing a large number of isoforms to experiments that utilize clinical-grade hAAT. Thus, the use of recombinant protein technology in the present experimental design facilitates an evaluation of molecularly uniform sequence-modified species of hAAT.

In the present study, we revisit a previously reported intra-RCL mutation (P357C); this mutation has been established as lacking anti-elastase activity, but was never evaluated for anti-inflammatory or immunomodulatory functions. Here, the functionality of a recombinant hAAT that bares this mutation is compared to an amino acid switch at the same position (P357A). Per our findings, both variants were compared to recombinant hAAT formulation that bare a mutation outside the RCL: cysteine 232 was replaced with proline (C232P).

As expected, disruption of the primary sequence of the RCL indeed nullified elastase inhibition. Interestingly, the mutation *outside* the RCL, C232P, also resulted in lack of elastase inhibitory capacity. Based on structural prediction of naïve hAAT, it is noted that position 232 is relatively proximal to the RCL and thus may partake in the conformational changes associated with protease binding. Nevertheless, it represents a variant of hAAT that, like PC and PA, fails to inhibit elastase and is thus of interest for studying elastase-independent anti-inflammatory and immunomodulatory molecular aspects of hAAT.

Similar to plasma-derived hAAT, the anti-inflammatory qualities of recombinant WT-hAAT have been established in other studies (26, 34, 36, 68). However, in the present study, variations surfaced in the anti-inflammatory potency between recombinant variants of hAAT, most notably in the case of the extra-RCL mutant, CP. In this mutated recombinant form of hAAT, greater anti-inflammatory effects were consistently observed. For example, pretreatment of cells with CP-hAAT resulted in reduced supernatant levels of both TNFα and IL-6 at *lower* concentrations than those required by recombinant WT-hAAT, and in a concentration-dependent manner. Additionally, at a uniform concentration of 200 ng/ml of recombinant formulations of hAAT, CP-hAAT was more potent than the other formulations with regards to changes in cell activation markers, *in vitro*. *In vivo*, CP-hAAT treatment resulted in LPS-stimulated animals displaying 2.4-fold lower proportions of CD40^HI^ cells, than in the LPS-stimulated WT-hAAT-treated group.

While some mutations may result in reduced half-life time due to unexpected binding partners, as well as changes in distribution volume and altered susceptibility to proteolytic attacks, the study of hAAT variant pharmacokinetics indicates that WT, PC, and PA share similar properties. In contrast, CP presented with a larger distribution volume and an extended half-life. While these results increase our confidence that the novel variants share other pharmacokinetic qualities to WT, the possibility of unique binding partners should be addressed in future studies.

In regard to CP increased distribution volume and half-life, since these distinctions appear to accompany this variant’s enhanced anti-inflammatory capacity, it is suggested that its physical properties render it functionally unique. It has been shown that exogenously administered hAAT is readily detected on the surface of activated immune cells (78), coinciding with observations by Subramaniyam et al., in which hAAT localizes on membrane lipid rafts (29). This property is in agreement with evidence for direct binding of oxidized cholesterol (31) and fatty acids (79) by hAAT, and suggests that hAAT membranal presence might serve as a platform for several of its functional attributes (80–82). Considering the abrupt structural change that a proline is predicted to exert at position 232, it is possible that the structural properties of CP allow it to better adhere to cell membranes, accounting for the rapid decline in serum levels of its soluble form on the one hand, and its significantly prolonged half-life on the other. Given that CD40 and CD86 signal transduction requires surface di- or -trimerization events, and that TNFα release is dependent on an intact surface protease, it is possible that increased membranal presence of CP might disrupt these processes. Specific studies are required in order to confirm this hypothesis.

Particular attention was relegated to TNFα in the present study. TNFα is one of the most consistent responders to hAAT treatment across inflammatory models (4, 83). Given that soluble TNFα levels are the result of transcription, expression, and proteolytic shedding processes, we sought to investigate the mechanisms by which hAAT treatment facilitates a reduction in the inducible levels of soluble TNFα. In the present study, the effect of each of the rhAAT variants over TNFα transcript levels, as well as membrane-associated TNFα and soluble supernatant/serum levels, were assessed. Our findings show that, as expected, inducible serum TNFα levels are reduced under WT-rhAAT treatment *in vivo*, consistent with other studies (84, 85). However, the effect of the variants varied: PA treatment resulted in minimal changes compared to the non-treated group, while both CP and PC treatments resulted in a sharp decline in TNFα levels. Intriguingly, CP treatment resulted in an isolated spike in TNFα levels at the 90-min time-point. This latter finding is in accordance with a study by Janciauskiene et al., in which a brief treatment of human monocytes with hAAT *in vitro*, results in a short-lived *elevation* in TNFα, IL-1β and IL-8 levels (86), an overt inflammatory response. While pretreatment of peritoneal macrophages with any of the rhAAT variants indeed resulted in reduced soluble TNFα levels within 24 h, only two variants, WT and PA, resulted in a detectable change in inducible *membrane*-associated TNFα levels. Considering that IL-6 secretion is ADAM17-independent, it is interesting to note that exposure to increasing WT or CP concentrations, but not PC or PA, resulted in similar IL-6 and TNFα supernatant concentrations, hinting at a more elaborate anti-inflammatory mechanism than the otherwise anticipated anti-proteolytic aspect of hAAT. Regarding intracellular expression of TNFα and its transport to the cellular membrane, an unexpected outcome was observed; pretreatment of cells with either WT-rhAAT or any of the other three hAAT variants not only did not reduce TNFα transcripts levels, but, in the cases of WT-rhAAT and CP treated cells, TNFα mRNA transcript levels increased. Collectively, the findings suggest that the effect of hAAT on the production and shedding of TNFα from leukocytes may involve multiple structural domains on hAAT, altogether irrespective of the RCL, affecting both intra- and extracellular elements in TNFα levels. In support of this possibility, antithrombin III, which shares structural homology to hAAT outside the RCL, has been shown to produce several overlapping outcomes to some of those obtained using hAAT (87, 88), including reduced LPS-stimulated TNFα release in monocytes (89).

While the majority of structural studies on hAAT focus on AATD-related aspects of the molecule, such as the occurrence of naturally occurring mutations, their potential to aggregate and their anti-proteolytic qualities, our data suggest that the non-RCL sections on the generous surface of hAAT may be of relevance to novel modifications for the purpose of enhanced functionality. Future potentiated variants of hAAT with longer half-life may be pharmaceutically attractive in as far as the renowned low patient compliance to repeated i.v. infusions, potentially offering future recombinant regimen that achieves the desired outcomes of hAAT at several-fold lower concentrations of the molecule. As such, one of the major limitation of introducing large volumes of hAAT may be lifted, promoting the exploration of subcutaneous hAAT treatment in humans.

Further studies are required in order to fully explore the immunological effects of the described structural variations of hAAT, including their potential clinical applicability for employing specific enhanced qualities of hAAT, such as half-life or anti-inflammatory potency. The outcomes hereby, once further investigated and developed, may be translated into the design of context- and disease-oriented therapeutic hAAT variants.

## Ethics Statement

All experiments involving animals conducted in this study were approved by the Ben-Gurion University of the Negev Animal Care and Use Committee.

## Author Contributions

YL: protein design, *in vitro* and *in vivo* immunological experiments design, execution and analysis, and writing. MZ: protein design and fabrication and purification. DO: protein design, *in vitro* and *in vivo* immunological experiments design, execution, and analysis. DL: protein fabrication and purification. BB: *in vitro* and *in vivo* immunological experiments execution. RS: *in vitro* and *in vivo* immunological experiments execution. AA: protein design and fabrication and purification, and writing. EL: mentoring, experimental design and analysis, and writing.

## Conflict of Interest Statement

All authors declare no personal, professional, or financial conflict of interest present at the conduction of this work.
